# Occupational Dust Exposure and Respiratory Protection of Migrant Interior Construction Workers in Two Chinese Cities

**DOI:** 10.3390/ijerph191610113

**Published:** 2022-08-16

**Authors:** Jinfu Chen, Bowen Cheng, Wei Xie, Min Su

**Affiliations:** 1 Department of Pathology, Guangdong Provincial Key Laboratory of Infectious Diseases and Molecular Immunopathology, Shantou University Medical College, Shantou 515041, China; 2 MPH Education Center, Shantou University Medical College, 22 Xin Ling Road, Shantou 515041, China

**Keywords:** migrant construction workers, dust exposure, respiratory protection level

## Abstract

Migrant interior construction workers are increasing in China. Construction workers are at an increased risk of work-related illness (WRI) due to prolonged exposure to and inhalation of dust. Dust concentrations in the air can be reduced significantly with effective respiratory protection measures. We assessed the dust exposure and factors associated with respiratory protection of migrant interior construction workers. The total dust concentration in the workplace ranged from 0.07 to 335.27 mg/m^3^, with a total dust exceedance rate of 50.00%. The respiratory dust loading ranged from 0.03 to 220.27 mg/m^3^, with a respiratory dust exceedance rate of 71.42 %. The highest total dust concentration occurred when masons were polishing cement walls. We performed a questionnaire survey of 296 persons in two cities in China, in which 87.84% had no respiratory protection or only one protection measure. Gender, workplace, respiratory disease, and protective attitude all had an effect on the level of respiratory protection. The dust exposure in most jobs exceeds hygiene standards. The respiratory protection of migrant interior construction workers in China is inadequate.

## 1. Introduction

With the growing urbanization of China and quick economic growth, the real estate industry has developed significant economic potential, and investment has increased. The houses developed by real estate and built for sale or rental in the market are called commercial houses. According to data released by China’s National Bureau of Statistics, real estate development businesses sold around 13.56 million housing units in 2020.

With the improvement of people’s living standards, residents have higher requirements for the decoration concept of their house, making it personalized and practical. The decoration of new apartments and remodeling of old apartments increases the demand for interior construction workers. According to a certain design scheme, some walls and water and electricity pipes formed during the civil construction stage are changed, and floors, walls and countertops are covered with wood, tile or marble, and painted.

Migrant workers mainly refer to workers who migrate from rural areas to cities and towns to engage in non-agricultural work. With the continuous acceleration of the new urbanization process, the number of migrant workers continues to rise, and they make significant contributions to the development of cities. However, they generally have low education, unstable jobs, low wages, and limited access to health service utilization [1,2]. Most of the people engaged in interior decoration are migrant workers [3,4]. They know little about the hazards in the work environment and lack self-protection awareness. Dust consists of solid particles ranging in size from below 1 µm up to around 100 µm [5]. Dust emitted from construction and renovation works is one of the most serious occupational hazards in the construction branch of industry. Silica is found in concrete, bricks, tiles, man-made stones, cement, and other building materials. Wood processing is always accompanied by the generation of wood dust, which is classified as a human carcinogen [6]. The construction environment for interior decoration is essentially in a semi-closed state. Once the dust is generated, it is difficult to remove, increasing the dust concentration in the air. Whether or not an airborne particle is inhaled depends on its aerodynamic equivalent diameter (AED), the velocity of the surrounding air, and the persons’ breathing rate [5]. Long-term exposure to and inhalation of dust puts construction workers at an elevated risk of work-related illness (WRI), particularly respiratory disorders such as pneumoconiosis, asthma, and lung cancer [7,8,9]. There is no obvious symptom in the early stage of the disease, so it is not easy to be diagnosed in time, but the resultant pulmonary fibrosis is irreversible. In the Dutch Zutphen study, an increased risk for nonspecific lung disease was found among construction and cement workers [10].

The risk of silicosis in the construction industry is significant, but it is often not recognized. As early as 1991, it was discovered that Scottish masons exposed to inhalable quartz for an extended period perished of accelerated silicosis. Although workers were aware of the health dangers associated with quartz at the time, they were unable to convince managers to offer adequate preventative measures and actions until significant disease developed [11]. Approaches to silicosis in Western Europe have mainly focused on mining, making silicosis even more invisible elsewhere, despite well-known hazards in other activities. Due to the lack of comprehensive and sensitive health monitoring system to prevent and detect silicosis (and other possible related diseases), silicosis is likely to be ignored [12]. In recent years, silicosis caused by the use of artificial stones with high-silicon content to make benchtops usually develops rapidly [12,13]. To avoid a repeat of this disaster, authorities around the world, including China, have implemented a variety of safety measures, including dust masks, local exhaust ventilation, and wet operations. These techniques help reduce the amount of dust breathed by employees, consequently reducing chronic respiratory symptoms and the prevalence of COPD, as well as improving lung function [14,15,16,17,18].

With regard to the health of migrant workers, much attention has been paid to the risk of communicable diseases [19], but less attention has been paid to non-communicable diseases, especially occupational exposures. Current studies of dust-related occupational hazards among migrant interior construction works in China are few. Therefore, we studied the dust exposure of different work types and the influence of different factors on the level of respiratory protection. This enriches the research on dust exposure and protection of construction workers and helps workers understand the hazards and the control of dust in the workplace, so as to prevent adverse effects.

## 2. Methods

### 2.1. Study Design

This was a cross-sectional study conducted between January 2019 and May 2020. Dust detection was conducted in Shantou, Guangdong Province, China. After interviews and pre-investigations, convenience sampling was used to determine the detection points, and dust detection was carried out after informed consent was obtained. The respiratory dust concentration and total dust concentration were measured simultaneously. According to GBZ/T 159-2004 Specifications of air sampling for hazardous substances monitoring in the workplace and GBZ/T 192.1-2007 Determination of Dust in The Air of The Workplace, filter membranes were used to sample dust. The sampling method used was a fixed-point, short-time, high-flow sampling. Each sampling point was sampled for 15 min, and the sampling flow is 20 L/min. The sampling point was set at about 1.5 m near the working site where dust was emitted. It is generally believed that dust particles with AED of less than 15 µm can enter the respiratory tract. Dust particles below 5 µm can reach the deep respiratory tract and alveolar area, which is called respiratory dust. The respiratory dust concentration and total dust concentration were calculated by using the mass of filter membrane and glass trap plate before and after sampling.

The respiratory protection level was investigated in migrant interior construction workers in Shantou City, Guangdong Province, China, and Qingyang City, Gansu Province, China. According to the type of work, the workers are mainly divided into masons, water electricians, carpenters, painters and general workers.

Our study adopted convenience sampling. After obtaining informed consent from the research subject, the investigator conducted a face-to-face interview and filled in the questionnaire. The questionnaire included the following parts: (1) basic information, which was divided into demographic characteristics and respiratory health status, (2) on-the-job situation, (3) use of daily protective measures, and (4) protection knowledge and protection attitude.

The respiratory protection survey used the cross-sectional survey sample size calculation formula. Generally, the utilization rate of dust-proof equipment for construction workers is approximately 20% [20], which required a total sample size of 283 considering the response and sampling error. A total of 315 workers were surveyed in this study, and 296 were finally included in the analysis.

$$n=\frac{Z_{1-\propto /2^{2}p\left(1-p\right)}}{d^{2}}$$

This study was reviewed and approved by the Ethics Committee of Shantou University Medical College (ethical clearance approval number SUMC-2018-59).

### 2.2. Statistical Analyses

SPSS22.0 was used to conduct the statistical analysis. The measurements of dust concentration were compared to current binding health standards, and the percentage of exceedances was estimated. Proportions were used in descriptive statistical analysis. A Wilcoxon rank-sum test was used to determine if the differences in composition ratios between groups were statistically significant. Due to the outcome incidence (respiratory protection) of over 10%, Poisson generalized loglinear model was used to examine the factors associated with respiratory protection for migrant interior construction workers.

## 3. Results

### 3.1. Dust Exposure

**Figure 1:**
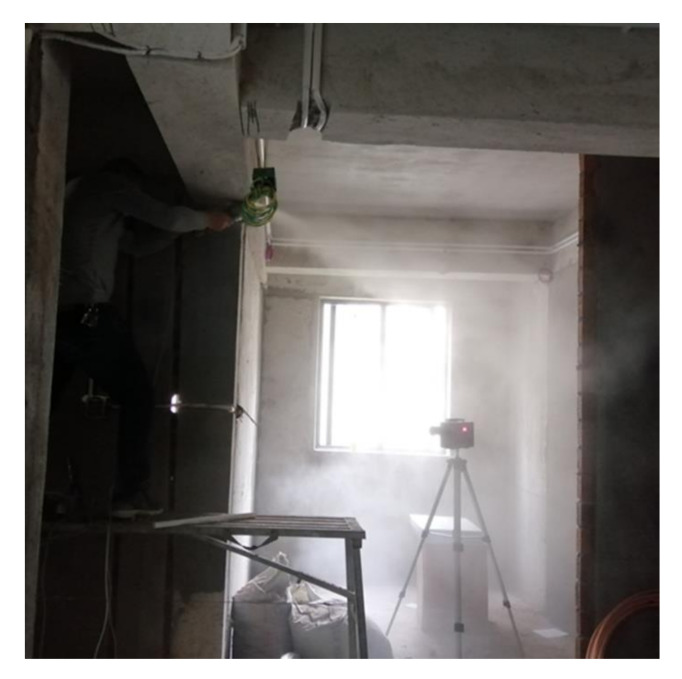
Dust detection carried out in the interior construction site.

The majority of construction workers on interior sites are exposed to visible dust (Figure 1). Dust exposure detection was carried out on masons, water electricians, painters, and carpenters. In view of the voluntariness of the participation and the different ongoing working activities of each specific construction site, the number of samples for the four work types was different. A total of 34 total dust detection values and 35 respiratory dust detection values were finally obtained. The sample sizes for masons, water electricians, carpenters, and painters were 12, 6, 11 and 5, respectively. Table 1 shows the measurements and exceedance standards for the four work types. The total dust concentration in the workplace ranged from 0.07 to 335.27 mg/m^3^, with a total dust exceedance rate of 50.00%. The respiratory dust loading ranged from 0.03 to 220.27 mg/m^3^, with a respiratory dust exceedance rate of 71.42%.

Based on the sample classification and permissible concentrations of dust in the air of the workplace (Supplementary Table S1), there is no statistically significant difference in the exceedance standard rate of total dust and respiratory dust among the four types of work. When comparing the excursion limit of each type of work, the total dust for carpenters and painters had statistical difference (Table 1).

### 3.2. Respiratory Protection Level

#### 3.2.1. Basic Situation

There were 296 questionnaires analyzed. The average age of the survey subjects in this study was 41.20 ± 11.09 years old, including 265 males, with a male-to-female ratio of 8.55:1. Regarding the use of respiratory protection measures, the number of people wearing dust masks during construction was 158 (53.38%), the number of people working in water sprinkling/wet operations was 38 (12.84%), and the number of people using suction/blower during the operation was 26 (8.78%). Based on the above three types of respiratory protection, construction workers having no protection, only one protection, any two kinds of protection, or all three kinds of protection were classified as none (117 persons (39.53%)), bad (143 (48.31%)), general (29 (9.80%)), and good (7 (2.36%)). There were statistical differences in the proportion of the respiratory protection level for different genders, workplaces, exposure to second-hand smoke at work, respiratory symptoms in the last three months, and respiratory diseases (*p* < 0.05) (Table 2). Of the masons, 47.83% were using masks, 27.83% were working in water sprinkling or wet operations, and 13.04% were using suction or blowers. Water electricians used masks in 68.85% of cases, 6.56% were in sprinkling/wet operations, and 3.28% in suction/blower operations. 32.08% of carpenters used masks, 1.89% worked in water sprinkling/wet operations, and 7.55% used suction/blower. 68.00% of the painters were wearing masks, none were performing water sprinkling or wet operations, and 8.00% were using suction/blower. Of the general workers and others, 58.82% were using masks; 5.88% were working in water sprinkling or wet operations; and 5.88% were using suction or blowers.

#### 3.2.2. Occupational History and Protective Attitude

The working years of construction workers spanned ≤10 years (99, 33.45%) and 11–20 years (106, 35.81%). There were 199 (67.23%) construction workers who had irregular working hours per day. The current employers of migrant interior construction workers were mostly contractors (217, 73.31%). Only 53 (17.91%) of the workers surveyed had undergone pre-job physical examinations, and the respiratory protection level of pre-job physical examinations was statistically significant (*p* < 0.05). Of those who had a pre-job physical examination, 69.81% had a bad level of respiratory protection, while 20.75% used no RPE (Table 3).

In this protective attitude study, few construction workers (31, 10.47%) answered that there were protective measures in the construction industry, and the rest answered no (182, 61.49%) or unclear (83, 28.04%). The respiratory protection level of protective attitude is statistically significant (*p* < 0.05). Among the renovation workers, 91.89% answered that the employer had not explained the dust hazards and available protection, 79.5% believed that there was a large amount of dust in the working environment, and only 33.45% believed that it was necessary to improve education regarding dust hazards and protection (Table 3).

#### 3.2.3. Analysis of Factors Related to Respiratory Protection Level for Migrant Interior Construction Workers

The following statistically different variables were included in the Poisson generalized loglinear model: gender, workplace, respiratory symptoms in the last three months, respiratory diseases, pre-employment physical examination, whether workers knew that there were protective measures and regulations in the construction industry, whether employers explained dust hazards and protection, whether workers thought there was a large amount of dust in the working environment, and whether workers thought that they needed to be educated about dust hazards and protection (nine variables).

Males had worse respiratory protection compared to females (*OR*: 1.28, 95% *CI*: 1.16–1.40). There was better respiratory protection in Shantou compared to Qingyang (*OR*: 0.83, 95% *CI*: 0.76–0.90). There was better respiratory protection for those with respiratory disease compared to those without respiratory disease (*OR*: 0.80, 95% *CI*: 0.68–0.94). Respiratory protection was better for those who knew about protective measures and regulations than for those who did not know about them (*OR*: 0.79, 95% *CI*: 0.65–0.97). Respiratory protection was considered to be better for those who were aware of large amounts of dust in the working environment (*OR*: 0.84, 95% *CI*: 0.79–0.90). Respiratory protection was better for those who thought they needed to be educated about dust hazards and protection (*OR*: 0.88, 95% *CI*: 0.80–0.96) (Table 4).

## 4. Discussion

Migrant workers in China will not leave their country, which is different from international migrant workers, and migrant workers always work long hours, including weekends without paid leave. In China, there are relevant laws and regulations that require workers to obtain occupational safety and health services. China has taken a series of measures to reduce occupational hazards. The Law on Prevention and Control of Occupational Diseases and the Work Safety Law are two important laws and regulations. The Chinese Government prioritized occupational health in the action plan of Healthy China 2030 as one of its 15 major health projects, but it still cannot meet the needs of effective prevention and control [21,22,23]. In addition, the workers themselves do not always follow safety rules, choosing not to wear PPE because it does not fit or because they think the equipment interferes with their work.

In this study, we detected the dust concentration in the working environment of interior construction workers. Carpenters in our study all exposed to carcinogenic wood dust which should be kept at the lowest level in workplaces. Respiratory dust exposure levels for carpenters and painter are different. Dust detection is one of the most important measures to control dust concentration in the working environment and protect workers’ health. Different types of workers are exposed to different levels of dust, but the concentration often exceeds hygiene standards. Exposure to cement dust can cause various acute and chronic respiratory diseases, including respiratory function impairment. One study found that long-term exposure to cement dust significantly reduced lung function [24]. Wood dust has been classified in group 1 as carcinogenic to humans [25]. Studies have shown that reduced exposure to wood dust may also reduce respiratory symptoms [26]. Carpenter often use woodworking machines indoors without local exhaust or general ventilation. The water electricians install some simple circuits and water channels at the construction site. They also need to knock out some of the walls and line installation. Therefore, there is a need for effective interventions to reduce or prevent occupational dust exposure.

In the respiratory protection level survey, 87.84% of the workers had no respiratory protection or only one protection measure. This result is consistent with a survey to assess personal protective equipment use among building construction workers in Ethiopia [27]. Our research shows that the factors related to the level of respiratory protection of migrant interior construction workers are gender, workplace and respiratory disease. Construction workers knew about protective measures and regulations and their attitude toward dust protection also affected their own level of respiratory protection. Workers with respiratory illnesses are more aware of their protection at work. Many workers feel that it does not matter whether they need to learn about dust hazards and protection. The possible reasons are that peasant interior decorators are less educated, do not care about learning relevant knowledge, and overestimate their physical abilities. They are more concerned that the loss of income will affect their ability to support their families.

Men have a higher odds of bad respiratory protection than women, which may be connected to the fact that women are more sensitive to occupational dangers [28]. Migrant interior construction workers in economically developed areas have better levels of respiratory protection than in economically underdeveloped areas [29]. Shantou City is a special economic development zone in China and has a more developed economy. Qingyang City is located in an underdeveloped area in the northwest. The two cities have large differences in medical and health care facilities [30,31]. Shantou has lower odds of general respiratory protection, and its migrant interior construction workers have good protection.

Our research found that when construction workers believe that there is a large amount of dust in the working environment, they have lower odds of bad respiratory protection. They also had better protection when they knew about dust protection measures and regulations. While construction workers believe that it is necessary to improve knowledge about dust hazards and protection, they have higher odds of general respiratory protection. This shows that a correct understanding of the hazardous factors of the working environment and a positive protective attitude will have a positive impact on protective behavior. Publicity and education and occupational health training will help to improve workers’ awareness of occupational protection and change their protection behavior [32,33,34]. In our research, most workers do not understand occupational laws and regulations; some workers do not know the benefits of occupational legislation, and lack knowledge of occupational laws. This can be attributed to the low level of education and lack of regular training in the workplace [35]. The awareness of occupational health and safety of migrant interior construction workers should be improved [36].

This study provides some references for the occupational health content of dust-related aspects of migrant interior construction workers. Given that dust exposure exceeds occupational safety limits at many work sites, it is particularly important to wear dust masks correctly, while we should also actively explore other suitable dust prevention measures, popularize the knowledge of dust hazards and protection for workers, and implement standardized management.

### Strengths and Limitations

There are several advantages to our research. First, we focus on migrant interior construction workers, an increasingly big population that is gravely damaged by dust. Second, most exposure surveys are conducted for factories, and there are no such surveys for interior construction workers.

This study also has some limitations. This was a study conducted between 2019 and 2020, a relatively long period of time because the study subjects were not easily available and the impact of the COVID-19 outbreak was significant. The sample size for each worker’s dust exposure level testing was limited, and this study did not experimentally analyze the amount of silica in dust. The study uses a questionnaire to obtain information, which can easily be biased by self-reporting behavior. In addition, the actual exposure level of workers may be different from the workers’ self-perception.

## 5. Conclusions

In the construction work environment, the dust excess rate of detection points is relatively high, and the dust concentration excess multiples of some operating points far exceed the occupational health standards. The utilization rate of workers’ respiratory protective measures is low. The workers’ gender, workplace, respiratory disease, knowledge of protection measures and regulations, and attitude toward dust protection are all related to their use of personal protection. Health education should be carried out, worker self-protection awareness should be improved, and occupational health protection should be strengthened, especially for migrant workers who face multiple barriers that are detrimental to their ability to protect themselves from workplace hazards.

## Figures and Tables

**Table 1:** Dust exposure and exceeding standard of different types of work.

Type of Work	Total Dust					Respirable Dust				
	Samples	Min–Max (mg/m^3^)	Median/IQR (mg/m^3^)	Exceeding Standard Points ^b^	Exceeding Standard Rate (%)	Samples	Min–Max (mg/m^3^)	Median/IQR (mg/m^3^)	Exceeding Standard Points ^b^	Exceeding Standard Rate (%)
Masons	12	0.48 to 335.26	7.35 (15.53)	6	50.00	12	0.17 to 220.27	1.47 (7.54)	5	41.67
Water electrician	6	0.93 to 101.70	28.78 (70.44)	4	66.67	6	0.50 to 51.13	9.77 (31.13)	4	66.67
Carpenter	11	0.07 to 42.30	4.03 (5.70)	3	27.27	11	0.03 to 34.70	1.17 (1.57)	11	100.00 ^a^
Painter	5	3.37 to 100.73	50.00 (72.10)	4	80.00	6	1.20 to 73.33	30.03 (52.02)	5	83.33
Total	34	0.07 to 335.27	7.35 (33.11)	17	50.00	35	0.03 to 220.27	2.03 (12.47)	25	71.42

^a^ Wood dust was classified as carcinogenic to humans by IARC. ^b^ The excursion limit of dust is twice that of the permissible concentration-time weighted average (PC-TWA).

**Table 2:** Basic information on migrant interior construction workers according to respiratory protection level.

Variables *n* (%)	None *n* = 117	Bad *n* = 143	General *n* = 29	Good *n* = 7
Gender *				
Male	112 (42.26)	121(45.66)	25 (9.43)	7 (2.64)
Female	5 (16.13)	22 (70.97)	4 (12.90)	0 (0.00)
Age				
≤20	0 (0.00)	6 (100.00)	0 (0.00)	0 (0.00)
21–40	48 (38.40)	66 (52.80)	10 (8.00)	1 (0.80)
41–60	64 (42.11)	64 (42.11)	18 (11.84)	6 (3.95)
≥61	5 (38.46)	7 (53.85)	1 (7.69)	0 (0.00)
Workplace *				
Shantou	26 (25.74)	45 (44.55)	23 (22.77)	7 (6.93)
Qingyang	91 (46.67)	98 (50.26)	6 (3.08)	0 (0.00)
Education level				
Primary school and below	53 (47.75)	49 (44.14)	6 (5.41)	3 (2.70)
Junior high school	48 (34.04)	73 (51.77)	18 (12.77)	2 (1.42)
High school or vocational high school	16 (40.00)	17 (42.50)	5 (12.50)	2 (5.00)
College degree and above	0 (0.00)	4 (100.00)	0 (0.00)	0 (0.00)
Smoking				
Never	27 (32.93)	44 (53.66)	11 (13.41)	0 (0.00)
Once	2 (2.22)	6 (66.67)	1 (11.11)	0 (0.00)
Now	88 (42.93)	93 (45.37)	17 (8.29)	7 (100.00)
Exposure to second-hand smoke at work				
Yes	91 (36.69)	131 (52.82)	20 (8.06)	6 (2.42)
No	26 (54.17)	12 (25.00)	9 (18.75)	1 (2.08)
Respiratory symptoms in the last three months *				
Yes	1 (6.25)	11 (68.75)	3 (18.75)	1 (6.25)
No	116 (41.43)	132 (47.14)	26 (9.29)	6 (2.14)
Respiratory disease *				
Yes	0 (0.00)	3 (42.86)	4 (57.14)	0 (0.00)
No	117 (40.48)	140 (48.44)	25 (8.65)	7 (2.42)

* *p* < 0.05 indicates that the difference is statistically significant.

**Table 3:** Occupational history and protective attitudes of indoor construction workers according to the level of respiratory protection.

Variables *n* (%)	None *n* = 117	Bad *n* = 143	General *n* = 29	Good *n* = 7
Type of work				
Masons	42 (36.52)	49 (42.61)	19 (16.52)	5 (4.35)
Water electricians	18 (29.51)	39 (63.93)	3 (4.92)	1 (1.64)
Carpenters	35 (66.04)	15 (28.30)	2 (3.77)	1 (1.89)
Painters	16 (32.00)	30 (60.00)	4 (8.00)	0 (0.00)
General workers and others	6 (35.29)	10 (58.52)	1 (5.88)	0 (0.00)
Working years				
≤10	34 (34.34)	58 (58.59)	7 (7.07)	0 (0.00)
11–20	45 (42.45)	51 (48.11)	6 (5.66)	4 (3.77)
21–30	26 (38.81)	25 (37.31)	14 (20.90)	2 (2.99)
≥31	12 (50.00)	9 (37.50)	2 (8.33)	1 (4.17)
Are the daily working hours fixed				
Yes	40 (41.24)	44 (45.36)	10 (10.31)	3 (3.09)
No	77 (38.69)	99 (49.75)	19 (9.55)	4 (2.01)
Current type of employer				
contractor	87 (40.09)	104 (47.93)	21 (9.68)	5 (2.30)
construction company	9 (29.03)	19 (61.29)	3 (9.68)	0 (0.00)
householder	21 (43.75)	20 (41.67)	5 (10.42)	2 (4.17)
Pre-job physical examination *				
Yes	11 (20.75)	37 (69.81)	4 (7.55)	1 (1.89)
No	106 (43.62)	106 (43.62)	25 (10.29)	6 (2.47)
Do you know that there were protective measures and regulations *				
Yes	5 (16.13)	12 (38.71)	10 (32.26)	4 (12.90)
No	78 (42.86)	90 (49.45)	13 (7.14)	1 (0.55)
Unclear	34 (40.96)	41 (49.40)	6 (7.23)	2 (2.41)
Does the employer explain the dust hazards and protection *				
Yes	4 (16.67)	14 (58.33)	2 (8.33)	4 (16.67)
No	113 (41.54)	129 (47.43)	27 (9.93)	3 (1.10)
Whether there was a large amount of dust in the working environment *				
Yes	69 (29.49)	130 (55.56)	28 (11.97)	7 (2.99)
No	48 (77.42)	13 (20.97)	1 (1.61)	0 (0.00)
Do you need to be educated about dust hazards and protection *				
Yes	18 (18.18)	61 (61.62)	15 (15.15)	5 (5.05)
No	25 (38.46)	35 (53.85)	5 (7.69)	0 (0.00)
It doesn’t matter	74 (56.06)	47 (35.61)	9 (6.82)	2 (1.52)

* *p* < 0.05 indicates that the difference is statistically significant.

**Table 4:** Factors related to respiratory protection level for migrant interior construction workers (95% Confidence Interval).

Variables	β	*p*	*OR*	95% *CI*
Male (ref. female)	0.24	<0.001	1.28	1.16–1.40
Shantou (ref. Qingyang)	−0.91	<0.001	0.83	0.76–0.90
Have respiratory disease (ref. no)	−0.23	0.006	0.80	0.68–0.94
I knew that there were protective measures and regulations (ref. do not know)	−0.23	0.021	0.79	0.65–0.97
There was a large amount of dust in the working environment (ref. no)	−0.17	<0.001	0.84	0.79–0.90
I need to be educated about dust hazards and protection (ref. does not matter)	−0.13	<0.001	0.88	0.80–0.96

## Data Availability

The datasets used and analyzed in the study are available from the corresponding author on reasonable request.

## Supplementary Materials

The following supporting information can be downloaded at: https://www.mdpi.com/article/10.3390/ijerph191610113/s1, Table S1: Sample classification and permissible concentrations of dust in the air of the workplace.
