# Supplementary material for: Occupational Dust Exposure and Respiratory Protection of Migrant Interior Construction Workers in Two Chinese Cities
Source: Int J Environ Res Public Health. 2022 Aug 16;19(16):10113. doi: 10.3390/ijerph191610113 (PMC9408467; doi:10.3390/ijerph191610113)
Supplement: Supplementary file 1 [file ijerph-19-10113-s001.zip › ijerph-1807018-supplementary.pdf]

Supplementary Table S1. Sample classification and permissible concentrations of dust in the air of the workplace.

Work type	Working procedure	Dust type	PC-TWA（mg/m^3）	
			Total dust	Respirable dust
Masons	Mixing cement	Cement dust	4	1.5
	knocking out walls	Cement dust	4	1.5
	cutting tiles	Silica dust (10%≤free SiO_2≤50%)	1	0.7
Water electrician	line installation	Cement dust	4	1.5
	knocking out walls	Cement dust	4	1.5
Carpenter	cutting and grinding	Wood dust	3	-
Painter	grinding and painting	Talc dust	3	1
